# Hemosuccus Pancreaticus: 15-Year Experience from a Tertiary Care GI Bleed Centre

**DOI:** 10.5402/2013/191794

**Published:** 2013-02-28

**Authors:** Ashwin Rammohan, Ravichandran Palaniappan, Sukumar Ramaswami, Senthil Kumar Perumal, Anand Lakshmanan, U. P. Srinivasan, Ravi Ramasamy, Jeswanth Sathyanesan

**Affiliations:** Institute of Surgical Gastroenterology & Liver Transplantation, Centre for GI Bleed, Division of HPB Diseases, Stanley Medical College Hospital, Old Jail Road, Chennai 600 001, India

## Abstract

*Background*. Hemosuccus pancreaticus (HP) is a very rare and obscure cause of upper gastrointestinal bleeding. Due to its rarity, the diagnostic and therapeutic strategy for the management of this potentially life threatening problem remains undefined. The objective of our study is to highlight the challenges in the diagnosis and management of HP and to formulate a protocol to effectively and safely manage this condition. *Methods*. We retrospectively reviewed the records of all patients who presented with HP over the last 15 years at our institution between January 1997 and December 2011. *Results*. There were a total of 51 patients with a mean age of 32 years. Nineteen patients had chronic alcoholic pancreatitis; twenty-six, five, and one patient had tropical pancreatitis, acute pancreatitis, and idiopathic pancreatitis, respectively. Six patients were managed conservatively. Selective arterial embolization was attempted in 40 of 45 (89%) patients and was successful in 29 of the 40 (72.5%). 16 of 51 (31.4%) patients required surgery. Overall mortality was 7.8%. Length of followup ranged from 6 months to 15 years. *Conclusions*. Upper gastrointestinal bleeding in a patient with a history of chronic pancreatitis could be caused by HP. All hemodynamically stable patients with HP should undergo prompt initial angiographic evaluation, and if possible, embolization. Hemodynamically unstable patients and those following unsuccessful embolization should undergo emergency haemostatic surgery. Centralization of GI bleed services along with a multidisciplinary team approach and a well-defined management protocol is essential to reduce the mortality and morbidity of this condition.

## 1. Introduction

Hemosuccus pancreaticus (HP) is a rare and potentially life threatening clinical entity and is described as bleeding from the ampulla of Vater via the pancreatic duct. It is the least frequent cause of upper gastrointestinal bleeding (1/1500) and is most often caused by chronic pancreatitis, pancreatic pseudocysts, or pancreatic tumors [1–3]. HP is often difficult to diagnose, partly because of its rarity and due to its anatomical location and also because the bleeding is often intermittent and cannot be easily diagnosed by esophagogastroduodenoscopy (OGDscopy) in the intermittent phase [1, 4]. The diagnosis is not always easy to establish and often a long period elapses between the onset of the first symptoms and the precise location of the source of bleeding [2, 4]. The objective of our study was to highlight the challenges involved in the diagnosis and management of HP and to audit our unit's HP protocol to effectively and safely manage this condition.

## 2. Materials and Methods

We retrospectively reviewed the records of all 51 patients who presented with HP over the last 15 years at our institution between January 1997 and December 2011. We noted demographic data, history, diagnostic features including symptoms, physical examination and time to diagnosis, investigations and therapeutic modalities, as well as follow-up data.

All patients were managed as per our department's algorithm. Following aggressive resuscitation, they were subjected to esophagogastroduodenoscopy and a Duplex scan. After hemodynamic stabilization, a CT angiogram followed by angiography ± angioembolization was done. In the event of continued destabilization or failed angioembolization, the patient was taken up for emergency surgery. Celiac trunk and superior mesenteric arteriography were performed transfemorally using a 4-F catheter. Images were obtained using the digital subtraction technique. Demonstration of contrast agent extravasation and/or vascular anomalies like pseudoaneurysm was an indication to attempt the use of a microcatheter and microcoils. The treatment technique included a superselective catheterization of the feeding artery, followed by endosaccular treatment of the pseudoaneurysm. Proximal and distal feeding artery occlusions were done. All operated patients had a clinical examination after 1 month and were kept on routine followup. Angioembolized patients had clinical examinations during the 6-month period and if a pseudocyst existed on US or CT scan, endoscopic or surgical therapy was planned. Persistently symptomatic patients with chronic pancreatitis were offered surgery when indicated.

## 3. Results

The sample included forty-three men and eight women. Mean age was 32 years (11–55 years). Most common presenting symptoms were worsening anemia and malena in forty-seven and forty-eight patients. Transfusion requirements ranged from 3 units to 12 units (mean-7 units). (Tables 1 and 2). Twenty-six had tropical pancreatitis, nineteen patients had a diagnosis of chronic alcoholic pancreatitis, five patients had acute pancreatitis, and one patient was diagnosed as having idiopathic pancreatitis (Table 3).

### 3.1. Diagnosis

Upper GI endoscopy showed the presence of blood in the duodenum in 26 of 51 (51%) patients. The remaining patients had normal UGI endoscopic findings. Abdominal ultrasonography with Doppler examination was performed in all patients and diagnosed aneurysmal bleeding in nineteen patients. Contrast-enhanced computed tomography (CECT) angiogram was performed in all 51 patients and showed a pseudoaneurysm in 46 (90%) patients. Selective angiography was done in 45 patients with a therapeutic intent (Table 4). The combined modality of CECT angiogram and conventional angiogram showed that the pseudoaneurysm was located in the head of the pancreas in 31 patients and in the body or tail in the remaining 20 patients. The mean (range) diameter of the pseudocyst was 67 (10–150) mm and the mean diameter of the pseudoaneurysm was 24 (7–80) mm. The pseudoaneurysm arose from the splenic artery in twenty-seven patients, from the gastroduodenal artery in nine, from a branch of the superior pancreaticoduodenal artery in two, from the inferior pancreaticoduodenal artery in two, and from the superior mesenteric artery in one patient. An unnamed vessel in the pseudocyst wall was the cause of bleed in nine patients. A ductal communication with the splenic vein was found in one patient. An arterial abnormality was found to be the principle cause in 50 of 51 (98%) patients (Table 5). 

### 3.2. Management

**Figure 1:**
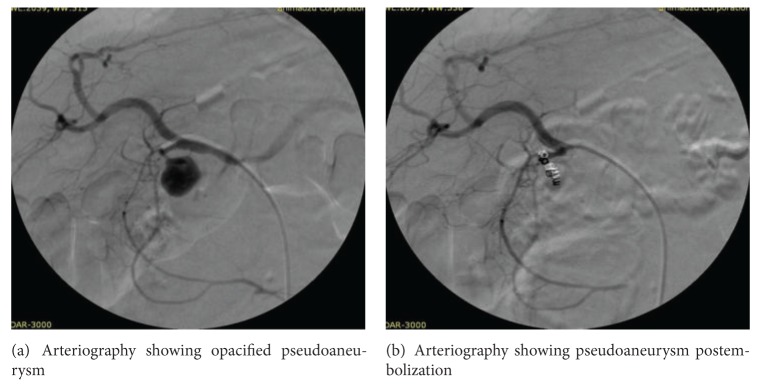
Hemosuccus pancreaticus—Angiographic Management.

**Figure 2:**
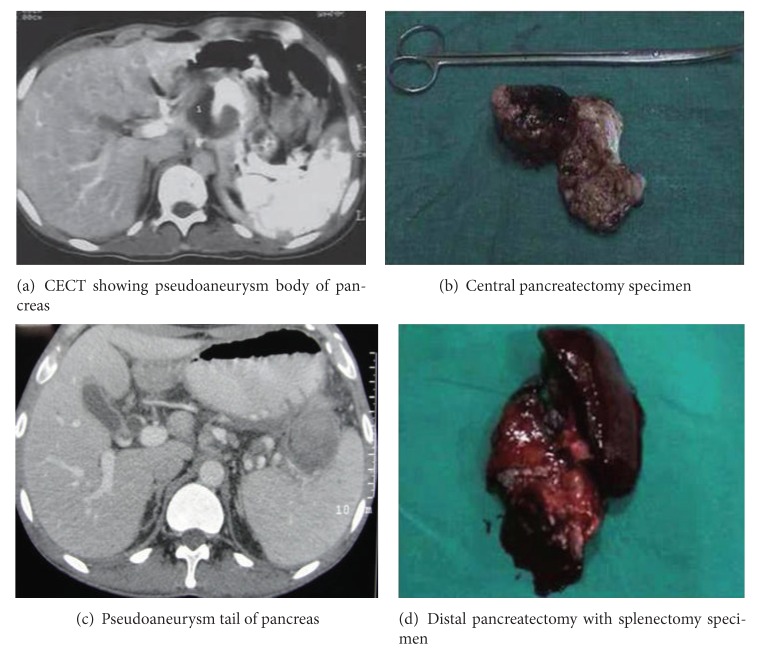
Hemosuccus pancreaticus operative management.

Selective arterial embolization was attempted in forty of forty-five (89%) patients and was successful in 29 (72.5%) patients (Figure 1). Sixteen of forty-five (36%) patients required surgery to control bleeding after the failure of arterial embolization in eleven and surgery in an emergent setting in five patients. Procedures included distal pancreatectomy and splenectomy in nine cases, central pancreatectomy in one case, intracystic ligation of the blood vessel in five cases, and aneurysmal ligation and bypass graft in one case (Table 6 and Figure 2). Six patients were managed without any therapeutic intervention apart from hemodynamic resuscitation, out of which four refused any form of therapy, and two patients exsanguinated before any therapeutic modality could be offered. There were four mortalities, one patient following surgery alone and one following surgery for a failed embolization. Two patients expired before any procedure could be performed. Morbidity included external pancreatic fistula in four patients (managed conservatively), ischemic cholecystitis (managed with percutaneous cholecystostomy) in one, wound infection in eleven patients, incisional hernia in three, and pneumonia in six patients (Table 7).

### 3.3. Followup

Length of followup ranged from 6 months to 15 years. Twelve patients were lost to followup. None of the thirty-five patients, who were followed up, experienced a recurrent bleed. Seven of the patients with chronic pancreatitis, who had undergone previous embolization, developed intractable pain and needed drainage procedures.

## 4. Discussion

Hemosuccus pancreaticus is a very rare cause of upper gastrointestinal bleeding. Approximately 150 cases have been reported in the literature since it was first reported by Lower and Farrell in 1931 [3]. In 80% of the cases, hemosuccus pancreaticus complicates an underlying pancreatic disease [5, 6]. Clinical symptoms and signs include UGI bleeding as evidenced by haematemesis and malena, of which malena is more common. Epigastric pain results from the elevation of pressure in the pancreatic ducts caused by blood clots [1, 5–7]. The haemorrhage is usually intermittent, repetitive and, most often, not severe enough to cause haemodynamic instability despite the usual arterial origin of bleeding [7, 8]. Other clinical signs are more exceptional and include jaundice, vomiting, weight loss, and a palpable pulsating mass with a systolic thrill in the event of aneurysm [1, 7–9]. Liver function test is normal apart from an increased serum bilirubin in the event of pancreaticobiliary reflux. Serum amylase is normal outside episodes of acute pancreatitis. It is difficult to diagnose HP because the bleeding is usually intermittent. Endoscopy is essential in ruling out other causes of upper gastrointestinal bleeding and in rare cases; active bleeding can be seen from the duodenal ampulla [9–11]. Even though endoscopy may be normal, it helps to rule out other causes of upper digestive bleeding (erosive gastritis, peptic ulcers, and oesophageal and gastric fundus varices, etc.) [8–10]. Ultrasonography can be used to visualize pancreatic pseudocysts or aneurysm of the peripancreatic arteries. Doppler ultrasound or dynamic ultrasound has been reported to be diagnostic. Contrast-enhanced CT is an excellent modality for demonstrating the pancreatic pathology and can also demonstrate features of chronic pancreatitis, pseudocysts, and pseudoaneurysms. On precontrast CT, the characteristic finding of clotted blood in the pancreatic duct, known as the sentinel clot, is seldom seen. Computed tomography may show simultaneous opacification of an aneurysmal artery and pseudocyst or persistence of contrast within a pseudocyst after the arterial phase. Again, these findings are only suggestive of the diagnosis. Ultimately, angiography is the diagnostic reference standard. Angiography identifies the causative artery and allows for delineation of the arterial anatomy and therapeutic intervention [9, 12–16]. HP is an entity diagnosed on clinical, endoscopic, and radiological findings, and a definitive diagnosis can be established only with angiography. Overall, the diagnosis of HP requires a high index of suspicion in patients with pancreatitis and GI bleeding. The natural history of chronic pancreatic pseudocysts and the risk of pseudoaneurysm formation are not well known [14–16]. The rate of pseudoaneurysm formation varies from 4% to 17% in operated pseudocyst patients and is about 7% in endoscopically treated series [17–19]. In a series of 14 patients with chronic pancreatitis and bleeding pseudocysts, 11 were treated successfully with embolization and 3 needed an operation. The overall mortality rate of 14% was related to the failure of the embolization or a complication. This compares favorably with the mortality rate of our series (7.8%). The authors concluded that arterial embolization is recommended as the initial therapeutic method, and further surgery should be reserved for patients in a good general condition who have other complications of chronic pancreatitis that need surgery [20]. Distal pancreatectomy for bleeding pancreatic pseudoaneurysms in the body or tail of the pancreas is a surgical alternative to angioembolization [21, 22]. When the pseudoaneurysm is located in the head of the pancreas, surgical resection is associated with increased mortality and morbidity, and angioembolization alone has been proposed as the recommended treatment modality of choice [19, 20, 23, 24]. Bleeding from the pancreaticoduodenal artery has a higher mortality rate than bleeding from the splenic or gastroduodenal artery (46% versus 21% and 28%, resp.) [23, 24]. Early angiography has halved the mortality rate. Once the haemodynamic situation is under control, interventional radiographic methods are used for initial treatment, with immediate good results in 60–100% of cases (72.5% in our series) [9, 15, 21]. Angiographic intervention of a haemorrhage from pseudoaneurysm in HP can be carried out either to stabilize the patient in order to perform elective surgery or as a definitive treatment [22, 23, 25]. Failure of catheter embolization may result from factors such as inability to isolate the bleeding vessel, spasm of the bleeding vessel, incomplete arterial occlusion, or misidentification of the bleeding vessel [21, 25, 26]. If a conservative transarterial approach is selected in a patient with chronic pancreatitis, the remaining diseased pancreas adjacent to the previously injured artery may be the source of reoccurrence of arterial injury and bleeding. With increasing expertise and the use of superselective angiocatheters, therapeutic embolization can serve as a definitive management strategy. Surgical treatment is indicated in uncontrolled haemorrhage, persistent shock, when embolization is not feasible or when embolization fails (continued or recurrent bleeding). It is also indicated in patients who have other indications for operative intervention (pseudocyst, pancreatic abscess, gastric outlet obstruction, obstructive jaundice, or incapacitating pain) and are otherwise appropriate surgical candidates [27]. In our series, 16 of 45 (36%) patients required surgery to control bleeding after the failure of arterial embolization in five cases and in an emergent setting in eleven. Arterial ligation is also effective, but it does not avoid the risk of recurrence. Drainage of the pancreatic pseudocysts associated with arterial ligation is particularly effective and is associated with fewer complications of infection and necrosis compared with aggressive surgery [21, 25–27]. More aggressive surgery with pancreatic resection enables the treatment of both the pancreatic and arterial diseases. Surgical procedures in our series included distal pancreatectomy and splenectomy, central pancreatectomy, intracystic ligation of the blood vessel, and aneurysmal ligation and bypass graft. In patients with chronic pancreatitis, pancreaticoduodenectomy or splenopancreatectomy are preferred by certain authors, but the problems of potential perioperative complications and postoperative pancreatic insufficiency should not be overlooked. Less radical approaches such as central pancreatectomy and intracystic ligation of pseudoaneurysm can be performed in place of pancreaticoduodenectomy. The documented success rates in most surgical series are in the range of 70–85%, with a mortality rates of 20–25% [9, 15–17].

## 5. Conclusion

Upper gastrointestinal bleeding in a patient with a history of chronic pancreatitis could be caused by HP. Diagnosis is based on investigations that should be performed in all patients, preferably during a period of active bleeding. Therapeutic options consist of selective embolization and surgery. All hemodynamically stable patients with HP should undergo prompt initial angiographic evaluation and if possible, embolization. Hemodynamically unstable patients and those following unsuccessful embolization should undergo emergency haemostatic surgery. A multidisciplinary team approach at a tertiary care centralized GI bleed centre with a well-defined protocol is indispensable in drastically reducing mortality and morbidity.

## Figures and Tables

**Table 1:** Demographics.

Demographics	
Mean age, years (range)	32 (11–55)
Male : female	43 : 8
Transfusion requirements, units (range)	7 (3–12)

**Table 2:** Presenting symptoms.

Presenting symptoms	*N* = 51
Haematemesis	16
Malena	48
Pain abdomen	31
Worsening anemia	47

**Table 3:** Etiology.

Etiology	*N* = 51
Tropical chronic pancreatitis	26
Alcoholic chronic pancreatitis	19
Alcoholic acute pancreatitis	05
Idiopathic pancreatitis	01

**Table 4:** Investigations.

Investigations, positive yield	*n* = 51
Upper gastrointestinal endoscopy	26/51 (51%)
Ultrasound and Doppler study	19/51 (38%)
CECT	46/51 (90%)
Selective angiography	40/45 (89%)

**Table 5:** Source of bleed.

Source of bleed	*n* = 51
Splenic artery	27
Gastroduodenal artery	09
Unnamed Intracystic artery	09
Sup. Pancreaticoduodenal art.	02
Inf. Pancreaticoduodenal art.	02
Superior mesenteric art	01
Superior mesenteric vein	01

**Table 6:** Management strategy.

Management strategies	(*n* = 51)
Angiographic embolization attempted	40/45 (89%)
Angiographic embolization successful	29/40 (72.5%)
Surgery	16/45 (36%)
Distal pancreatectomy and splenectomy	09
Central pancreatectomy	01
Intracystic ligation of blood vessel	05
Aneurysmal ligation and bypass graft	01
No therapeutic intervention (apart from hemodynamic resuscitation)	06

**Table 7:** Complications.

Complications	
External pancreatic fistula	4
Ischemic cholecystitis	1
Wound infection	11
Pneumonia	6
Incisional hernia	3
Mortality	4
